# Progressive Cyclic Oculomotor Nerve Palsy: An Update Focusing on Accommodation and Visual Acuity Changes

**DOI:** 10.7759/cureus.117295

**Published:** 2026-10-01

**Authors:** Michael S Salman, Elizabeth Carson, Ian H Clark

**Affiliations:** 1 Department of Pediatrics and Child Health, University of Manitoba, Winnipeg, CAN; 2 Department of Ophthalmology, University of Manitoba, Winnipeg, CAN

**Keywords:** cyclic accommodation, cyclic ptosis, cyclic pupillary spasm, oculomotor nerve (cn iii) palsy, visual acuity

## Abstract

Cyclic oculomotor nerve palsy is a rare and typically congenital disorder. It is characterized by a unilateral third cranial nerve palsy with involuntary periodic spasms that cause one or more of the following: upper eyelid elevation, miosis, accommodation, and contraction of one or more of the extraocular muscles innervated by the third nerve. We provide an update on a previous case report of a female with this disorder and highlight how its features evolved over a 13-year follow-up period. Our patient presented in early infancy with a congenital partial right oculomotor nerve palsy without ptosis, which was later followed by cyclic spasms of the pupil. Cyclic accommodation was suspected at age five years because her visual acuity (VA) changed between the spastic and paralytic phases. At age 11 years, she developed cyclic ptosis. The periodicity of the spastic and paralytic phases varied over the years and from cycle to cycle. In addition, the spasms were usually not synchronous among the affected muscles within any single cycle. The pathogenesis remains unknown. Based on this single case report, we conclude that congenital cyclic oculomotor nerve palsy causes weakness in one or more muscles innervated by the oculomotor nerve, and this weakness may occur sequentially. The age of onset of spasms varies. The periodic spasms may affect one muscle at onset and may then involve additional muscles over subsequent years. The duration of the spasms varies across different muscles during any one cycle, across successive cycles in any one muscle, and over the years in any one muscle.

## Introduction

Monocular cyclic oculomotor nerve palsy is a rare disorder. It typically presents at birth or in early infancy with paresis of one or more muscles, which may later progress to involve other muscles innervated by the oculomotor nerve at variable intervals [1-6]. In 1975, an extensive review summarized its involuntary biphasic features, namely, a paralytic phase and a spastic phase [5]. Recently, a review of 124 cases further characterized its features [6]. At baseline, there is paralysis of one or more muscles innervated by the oculomotor nerve, followed by periodic spasms that involve one or more of these muscles. There are no pauses between the paralytic and spastic phases, which alternate continuously [6]. Usually, the two phases are noted at presentation [1,2]. However, in some cases, there is a delay of months to years between the onset of paresis and the development of cyclic spasms [2,4-6]. It is a lifelong disorder [3].

The affected pupil is usually unreactive to light or a near target throughout the cycle [1,2,4]. In most cases, a dilated pupil and upper eyelid ptosis are present during the paralytic phase [4,6]. During the spastic phase, the pupil constricts, and the upper eyelid elevates [1,2,4]. Infrequently, some authors have commented on variations in visual acuity (VA) and the degree of accommodation that occurs during the paralytic and spastic phases [1,2,4]. Typically, the paralytic phase lasts for about one minute, and the spastic phase lasts about half as long. However, the duration of the full cycle (paralytic and spastic phases) varies markedly from cycle to cycle in any one patient and among patients, ranging from one to three minutes, with an average of 72 seconds [3,5-7]. The direction of gaze does not typically influence the duration of the cycle [1-3], although some studies have reported that voluntary abduction prolonged the paralytic phase, whereas adduction prolonged the spastic phase [2,5,6].

The rhythmic cycling is involuntary and persists throughout the day and during sleep [1,2,3,5,6]. General anesthesia can abolish the spastic phases [3,7]. Amblyopia and strabismus occur frequently [1-6]. The cyclic spasms involve the sphincter pupillae in all cases, the levator palpebrae superioris muscle frequently, and the medial rectus muscle less commonly [5]. The other cranial nerves are not involved [2].

There is a paucity of long-term prospective follow-up studies of these patients. We have previously reported a case of isolated cyclic spasm of the pupil in a toddler with congenital partial oculomotor nerve palsy [8]. In this follow-up study, we provide an update on the same patient, now aged 13 years. Our objectives were to describe the evolution of weakness in one or more muscles innervated by the oculomotor nerve and to bridge the knowledge gap by documenting additional findings, including visual acuity changes, cyclic accommodation, cyclic ptosis, and asynchronous cycling among the muscles involved over a 13-year follow-up period.

The patient and her mother gave their verbal consent. The mother gave written permission for the publication of the case report, including the figure. Ethics approval was granted by the Health Research Ethics Board at the University of Manitoba.

## Case presentation

Birth to 19 months

A four-month-old girl with an unremarkable medical history and uncomplicated pregnancy was referred for strabismus. Her initial case report described her clinical features in detail up to age 19 months [8]. Briefly, she had a right exotropia of 70-80 prism diopters (PD). There were adduction and elevation deficits of the right eye. Right inferior oblique muscle weakness was suspected due to the presence of incyclotorsion of the right eye on ophthalmoscopy. There was no ptosis, and the pupils were equal, round, and reactive to light. A congenital right oculomotor nerve palsy involving three extraocular muscles was suspected.

At age six months, cyclic spasms of the right pupil were observed. Brain MRI with thin cuts (0.8 mm) was normal. At age nine months, inferior rectus weakness became evident. In addition, amblyopia was suspected because she would maintain fixation with the right eye for only a few seconds. Part-time occlusion of the left eye was recommended. At age 11 months, intermittent, non-cyclic twitching of the right upper eyelid was noted, which occurred independently of newly acquired, intermittent, non-cyclic partial ptosis. At age 15 months, her partial right ptosis became constant (non-cyclic). A repeat brain MRI was normal.

At age 18 months, cycling of the right pupil consisted of constriction to a diameter of 2 mm for 30 seconds, followed by dilation to 4 mm for 30-60 seconds. Brief right upper eyelid twitches were noted at the onset of the spastic phase. The right pupil was no longer reactive to light. No cyclic spasms of the right extraocular muscles with paralysis were seen. Her neuro-ophthalmological and neurological examination was otherwise normal.

At age 19 months, she underwent strabismus surgery for approximately 60 PD of exotropia with a small right hypotropia (10 mm right lateral rectus recession and 8 mm right medial rectus resection). At age 22 months, VA was 0.60 logMAR in the right eye and 0.00 logMAR in the left eye using Cardiff Cards while wearing her glasses (+3.00 diopters sphere in the right eye and plano in the left eye), with a right exotropia measuring approximately 10 PD and a right hypotropia of similar magnitude.

Twenty months to 11 years

At age 38 months, she underwent right frontalis suspension for ptosis. At age five years, cyclic accommodation was evident. Between the ages of six and 11 years, using the LEA Symbols near chart, VA ranged from 0.24 to 0.38 logMAR during the spastic phase and from 0.40 to 1.2 logMAR during the paralytic phase. Using a distance Snellen chart, VA in the right eye ranged from 0.38 to 0.80 logMAR; however, the phases were not documented at the time of testing during these clinic visits. At age 11 years, cyclic spasms of the right eyelid were noted for the first time, with the palpebral aperture in central gaze cycling between 3 and 5 mm.

At last follow-up (age 13 years)

A Righton Retinomax K+screen autorefractor (Right Mfg. Co., Tokyo, Japan) was used to track changes in pupil size and refractive error over 270 seconds under dim room lighting. Readings were obtained from both eyes at 30-second intervals.

Pupil Size and Reactivity

For the affected right eye, the pupil size ranged from 6.2-6.8 mm during the paralytic phase, with a mean of 6.5 mm, and from 3.6-3.8 mm during the spastic phase, with a mean of 3.7 mm; the mean change was 2.8 mm. For the unaffected left eye, the pupil size ranged from 5.3-6.1 mm, with a mean of 5.8 mm, during both phases of the right pupil.

The right pupil did not react to direct or consensual light during either the paralytic or spastic phases. There was also no reaction to a near target and no afferent pupillary defect. There was no change in the size of the right pupil when fixating centrally or during right and left gaze during each of the paralytic and spastic phases; i.e., there was no evidence of reinnervation, which might cause synkinesis. The left pupil displayed normal reactions to light and accommodative targets and was not affected by the cycling phases of the right eye.

Accommodation Magnitude

The cylinder reading of the affected right eye remained relatively stable (within +/-0.25 diopters (D)) at +6.50 axis 90. The sphere for the paralytic phase measured -2.75 to -3.00 D, with a mean of -2.83 D, whilst the sphere for the spastic phase was -6.75 to -7.50 D with a mean of -7.15 D. Hence, the mean myopic shift, as measured with an autorefractor, was 4.32 D from the paralytic to spastic phases. This phase-dependent refractive shift is consistent with cyclic accommodative spasm. There was one outlier data point of -9.50/+7.50 x 80 with a 6.8 mm pupil. This reading did not match the rest of the data collected and is assumed to be an inaccurate measurement.

Objective cycloplegic refraction was performed for comparison and was found to be -0.50/+4.75 x 90 for the right eye. The Retinomax cylinder measurements for the left eye also remained relatively stable (within +/-0.25 D) at +0.75 x 95. The sphere range was -1.75 to -3.00 D, with a mean of -2.14 D. Cycloplegic refraction for the left eye was -2.50/+0.75 x 90. 

The accommodative near point was also measured using the Krimsky-Prince rule for each eye. Normal accommodation for a 13-year-old with this test (according to the Krimsky-Prince rule) is 13.1 D. The accommodative near point for the affected right eye in the paralytic phase was less than 3.3 D (lowest number on the rule) and 6.8 D in the spastic phase. This is an accommodative shift of at least 3.5 D. The accommodative near point for the left eye was found to be better than 15.8 D (highest number on the rule).

Visual Acuity

VA was measured in each eye in primary position, right gaze, and attempted left gaze, during the paralytic and spastic phases (Table 1). VA measurements in up- and downgaze were not performed for the right eye due to its limited vertical movements, but it was measured in the left eye. Right-sided amblyopia is evident. In general, though not consistently, right eye near VA was better during the spastic phase compared to the paralytic phase, while distance VA was better during the paralytic phase compared to the spastic phase on her last assessment (Table 1). Gaze direction did not alter VA in a consistent manner, but she had difficulties with sustaining different directions of gaze, which made VA measurements unreliable in eccentric gaze. 

**Table 1:** Visual acuity ^*^We attribute this change (which is opposite to what we anticipated, namely, near VA should be better during the spastic phase) or ^**^lack of change in best-corrected VA to the asynchronous occurrence of pupil and accommodative spasms (see text in discussion). ^***^Patient had difficulty maintaining left gaze; hence, the reduction in right-eye VA in left gaze was noted during the paralytic phase Best-corrected VA measured for the RE and LE at last assessment during the spastic and paralytic phases of the right pupil for near and distance fixation (logMAR) VA: visual acuity; RE: right eye; LE: left eye

	Near VA RE		Near VA LE		Distance VA RE		Distance VA LE	
Pupils	Paralytic	Spastic	Paralytic	Spastic	Paralytic	Spastic	Paralytic	Spastic
Central gaze	0.54^*^	0.60^*^	0.04	0.04	0.72	1.10	0.00	0.00
Right gaze	0.70	0.60	0.04	0.04	0.66	1.00	0.00	0.00
Left gaze^***^	1.00	0.70	0.04	0.06	0.94^**^	0.94^**^	0.00	0.00
Upgaze	-	-	0.04	0.04	-	-	0.00	0.00
Downgaze	-	-	0.04	0.04	-	-	0.00	0.02

Palpebral Aperture Size

The palpebral aperture at rest for the affected right eye during the paralytic phase was 4 mm in central, right, and left gaze (N.B. a right frontalis suspension had been performed at age 38 months, which improved her ptosis). In the spastic phase, it was 5 mm in central, 3.5 mm in right, and 5 mm in left gaze. By observation, the difference in the palpebral aperture between the two phases was 1-2 mm. The left palpebral aperture at rest was 10 mm in central, right, and left gaze. It was 10.5 mm in upgaze and 9 mm in downgaze. These measurements did not change during the paralytic and spastic phases of the right eye.

Duration of Cycles

The initial plan was to measure the durations of the cycles and their phases for 120 seconds, aiming to capture about three to five full cycles. The observation period was extended following a second visit to complete the assessment. Data sets from the two visits were combined, approximately doubling the observation periods. Ptosis was not assessed on the first visit due to the patient’s fatigue.

During the first assessment, two observers (MSS and EC) assessed her at the same time. One observer called out the changes observed in the pupil, and the second observer recorded the time with a stopwatch. For VA, the patient reported when her vision changed, and the times were marked by the second observer. The second assessment was videotaped. The video was reviewed on a few occasions. Durations of the phases were measured by marking the time each change in the pupil size and palpebral aperture took place. To investigate synchrony among the changes affecting the various muscles in any one phase, the recorded video of her assessment was reviewed for changes occurring during each of the phases.

The duration of the total cycles and each of their two phases were variable in the muscles involved during any one cycle. These durations also varied in sequential cycles for each of the muscles affected by the cyclic spasms. In addition, the onset of spasms in each of the muscles involved occurred asynchronously most of the time during any one cycle (Table 2).

**Table 2:** Dynamic ocular changes during the two phases ^¥^During the spastic phase of the first cycle, lessening of the ptosis due to levator contraction, miosis, and clearer vision occurred concurrently, a rare occurrence. ^§^Typically, only palpebral aperture change occurred consistently first during the spastic and paralytic phases in sequential cycles. ^*^The spastic phase always started with brief right upper eyelid twitches followed immediately by levator contraction. ^ψ^Duration was not measured, as the period of observation finished before the end of the third spastic phase Time of onset and duration of changes in right eyelid aperture, pupil size, and vision during sequential spastic and paralytic phases, observed concurrently amongst the muscles involved at the last assessment

Cycle	First cycle		Second cycle		Third cycle	
Phases	First paralytic phase	First spastic phase^¥^	Second paralytic phase	Second spastic phase	Third paralytic phase	Third spastic phase
(A) Right eyelid:						
Onset time of eyelid aperture change (seconds)	0^§^	28	46^§^	63^§^	79^§^	102^§^
Change and phase duration (seconds)	More ptosis (28)	Less ptosis^*^ (18)	More ptosis (17)	Less ptosis^*^ (16)	More ptosis (23)	Less ptosis*^ψ^
B) Right pupil:						
Onset time of pupil size change (seconds)	3	28	57	74	84	104
Change and phase duration (seconds)	Mydriasis (25)	Miosis (29)	Mydriasis (17)	Miosis (10)	Mydriasis (20)	Miosis^ψ^
C) Vision:						
Onset time of visual acuity change (seconds)	5	28	51	64	82	104
Change and phase duration (seconds)	Blurry vision (23)	Clearer vision (23)	Blurry vision (13)	Clearer vision (18)	Blurry vision (22)	Clearer vision^ψ^

Pupils: The right pupil was observed in central gaze for 250 seconds (seven cycles). The paralytic (mydriatic) phase ranged from 5-25 seconds with a mean duration of 16.9 seconds. The spastic (miotic) phase ranged from 10-29 seconds with a mean duration of 19 seconds.

Accommodation: The accommodative cycles of the affected right eye were recorded for a total of 275 seconds (seven cycles) to determine if there was a predictable pattern for the duration of the paralytic and spastic phases. The patient verbally reported when the change in her vision occurred, i.e., blurry versus clearer. The duration of the blurred paralytic phase ranged between 13-32 seconds, with a mean of 22 seconds. The spastic phase with the clearer vision ranged between 13-23 seconds, with a mean of 16.7 seconds. These accommodative spasms did not usually occur at the same time as the observed changes in the palpebral aperture or pupil.

Palpebral aperture: The changes in ptosis were observed for 120 seconds (three cycles). The more-ptotic phase (i.e., during the paralytic phase) ranged from 17-28 seconds with a mean of 22.7 seconds. The less-ptotic phase ranged from 16-18 seconds with a mean of 17 seconds.

Synchrony and the sequence of changes in each phase and sequential cycles

The onset of cyclic changes in the muscles involved was typically asynchronous. In addition, the sequence of changes in the muscles involved in each of the two phases during any one cycle and sequential cycles was generally inconsistent, except for the palpebral aperture change, which consistently occurred first. During the spastic phase, levator contraction was always preceded by right upper eyelid twitches (Table 2).

Ocular motility and other exam findings

The following ocular motility and strabismus findings have been affected by her right eye strabismus surgery at one year of age (details above). There was no change in the right ocular motility during the paralytic or spastic phases. Adduction was -3.5, abduction -2.5, downgaze -3, and upgaze -4. The left ocular motility was normal (Figure 1).

**Figure 1:**
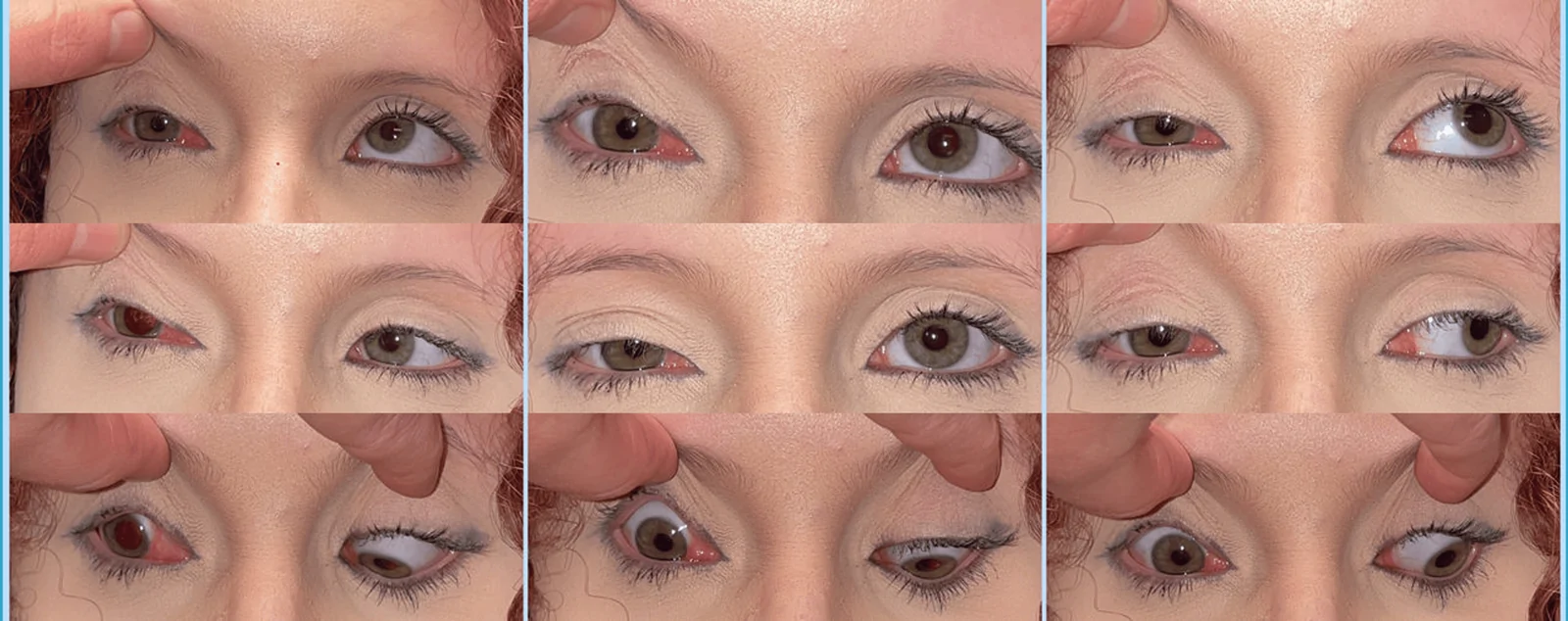
Representative photographs of the eyes in the nine cardinal positions of gaze Right third nerve palsy is evident. Note that the photographs in this image were not all taken during a single phase (either spastic or paralytic) due to the time required to capture the photographs and the relatively short periodicity and variable duration of these phases, so no comment will be made about right lid or pupil changes, which can be seen to be variable. In primary position, there is a small right hypotropia; the bulky and pink appearance of the nasal conjunctiva is the result of her large right medial rectus muscle resection. Left gaze shows almost no adduction of the right eye. Right gaze shows poor abduction due to the prior lateral rectus muscle surgical weakening; there may also be an element of right medial rectus muscle contracture contributing to her poor right abduction. Attempted upgaze shows no movement of the right eye. Attempted downgaze shows poor infraduction of the right eye. There were no deficits noted in the left eye

Strabismus was observed whilst performing an alternate cover test for 120 seconds: there was little movement from the primary position of the right eye, so the left eye was observed for a secondary deviation. The estimated findings are listed in Table 3. The strabismus varied throughout the observation period. 

**Table 3:** Strabismus findings Estimation of strabismus findings for near and distance fixation during the paralytic and spasm phases of the affected right eye; measurements in PD show the secondary deviation of the left eye PD: prism diopter

Viewing distance	Phase	Findings
Near	Spasm phase	Left hypophoria 12 PD in one phase. Small exophoria/hyperphoria in the next two phases
Near	Paralytic phase	Exophoric (at most 16 PD), left hypophoria (at most 5 PD)
Distance	Spasm phase	Small esodeviation (at most 4 PD), small left hyperphoria (at most 4 PD)
Distance	Paralytic phase	Exodeviation (at most 10 PD), left hypophoria (at most 5 PD)

Color vision, tested with Ishihara plates (Ishihara’s Tests for Colour Deficiency, concise edition 2005, Kanehara Trading Inc., Tokyo, Japan), was normal in each eye. Dilated fundus examination revealed healthy fundi with normal optic discs and normal maculae. The cup-to-disc ratio was 0.2 in both eyes, and no torsion was noted. 30-2 Humphrey visual field testing (Carl Zeiss Meditec AG, Jena, Germany) was normal for both eyes.

Facial sensation to touch and pinprick, jaw clenching, facial movements, corneal reflex, and hearing were normal bilaterally. Bell phenomenon was present on the left but absent on the right.

## Discussion

The evolution of congenital oculomotor nerve palsy and its cyclic spasms over several years of follow-up has not been documented in detail in prior case reports. However, in a few case reports, clinical features that developed over a short period of time have been mentioned [2,6,7].

The course of congenital oculomotor nerve palsy and its cycling spasms is progressive

Since dysfunction of some of the muscles innervated by the right oculomotor nerve in our case occurred following an earlier period of normal function, we suggest that this disorder is progressive. Indeed, our case eventually progressed from involving three extraocular muscles to involving all muscles innervated by the oculomotor nerve, each affected by a variable degree, followed by progressive cyclic spasms of some muscles innervated by the oculomotor nerve over time. First, our patient displayed weakness in three extraocular muscles in early infancy. This was followed shortly by the development of cyclic spasms of the pupil and then inferior rectus weakness. Subsequently, constant partial ptosis occurred, and there was loss of both the light reflex (direct/consensual) and miosis in response to a near target. Cyclic accommodation was first documented at age five years, when VA could be assessed more reliably; however, her age at onset is uncertain. It presumably resulted in right distance and near VA variations during her earlier clinical assessments. Cyclic spasms of the right eyelid developed at age 11 years.

Changes during the cycles

Pupil Size

The right pupil size during the two phases and the mean change in pupil size were moderate at 2.8 mm.

Accommodation

At her last assessment, the mean myopic shift was very significant at 4.32 D from the paralytic to the spastic phases. This was confirmed using both subjective (Krimsky-Prince rule) and objective (autorefractor) tests.

Visual Acuity

Our patient had variable right VA over the years, even after considering the phase in which it was measured. This variability is likely attributable in part to the development of amblyopia, the different methods used in measuring her VA over the years, and the variations in VA encountered on repeat testing. The favorable VA outcome was likely influenced by several factors: early recognition of her amblyopia and prompt institution of part-time occlusion; a significant improvement in her ocular alignment following strabismus surgery; her ptosis did not cover the pupil; and the fact that her involuntary periodic cyclic accommodation permitted clearer vision regularly.

Palpebral Aperture

The difference in the right palpebral aperture between the two phases was modest at best. However, the true difference in the palpebral aperture is unknown since the eyelid spasms first occurred many years after her ptosis surgery. A sling surgery in a young adult with cyclic ptosis diminished eyelid cycling likely because it bypassed levator function [9], which implies that our patient may have had a larger variation in her ptosis between the two phases if ptosis sling surgery had not been performed earlier. Furthermore, her sling surgery may have masked an earlier age of onset of her cyclic ptosis.

Duration of the cycles and their two phases

Duration of the full cycles and each of their paralytic and spastic phases varied over the years, including the cyclic pupil spasms that were documented as a toddler [8]. In addition, cycle-to-cycle variability was evident. The mean duration of accommodation and improved ptosis that occurred during the spastic phases were generally (though not consistently) shorter, whilst the duration of pupil spasm was generally slightly longer in comparison to the duration of the paralytic phase. The reason for this pattern of variability is unknown.

Variability in the duration of pupil phases from cycle-to-cycle has been documented previously [5-7]. Interestingly, the pupil spastic phases in our case were the same or shorter during infancy in comparison to the paralytic phases [8], a more common finding in many reported cases [5-7].

Asynchrony amongst muscles affected by the cyclic changes and their variable onset

The change in palpebral aperture, pupil size, and accommodation did not typically occur synchronously during either of the two phases. In addition, the sequence of changes in the muscles involved during any one phase or subsequent phases was not consistent, except for right upper eyelid twitches always heralding the onset of the spastic phases of the levator muscle, as reported previously [2,3,4,6,7]. We found no papers that described in detail the sequence of the changes seen in the muscles involved during each of the two phases or their respective phase durations.

We speculate that the observed (and mostly asynchronous) changes in the onset and variable durations of the phases in each of the muscles involved imply that there is more than one generator for the cyclic spasms, each starting at a different age and with an independent duration of the cyclic spasms. The location of these generators is presumed to be in the peripheral rather than the proximal branches of the oculomotor nerve supplying the muscles involved in the cyclic spasms, since the onset of the spasms does not usually occur synchronously during any one cycle. However, this hypothesis remains speculative and does not exclude central mechanisms. Alternative explanations for the asynchrony include differential excitability or recruitment of individual oculomotor nerve branches, different conduction properties, differing muscle contractile properties, mechanical factors, or separate central/ peripheral mechanisms.

We anticipated a priori that the onset of miosis or mydriasis would be synchronous with the changes in accommodation; hence we measured VA after the onset of miosis and mydriasis. However, this proved not to be the case. We believe that the asynchronous onset of the accommodative spasm and miosis likely resulted in the inconsistency in our VA observations (Table 1); i.e., an improvement of near VA during miosis and an improvement in distance VA during mydriasis was not consistently observed.

Absence of clinically demonstrable denervation-reinnervation synkinesis

The direction of gaze during the paralytic or spastic phases did not change VA or the size of the pupil of the right eye, consistent with the absence of denervation-reinnervation synkinesis, which may occasionally occur with cyclic oculomotor nerve palsy on attempted voluntary gaze change [5,6].

There was no change in the ocular motility of the right eye during the paralytic or spastic phases, but there was a change in the phoric deviation, as measured with the alternate cover test observing the left eye for a secondary deviation. While this would not represent any synkinesis, it does likely reflect a change in tonic drive to the right eye’s extraocular muscles innervated by the third nerve as part of this cyclic disorder.

Proposed mechanisms for the cyclic spasms

The mechanism underlying cyclic oculomotor paresis remains unknown. A few theories have been proposed. First, a central lesion affecting the intracranial portion of the oculomotor nerve with retrograde degeneration and reorganization of synaptic input to the oculomotor neurons [5]. Second, a peripheral oculomotor nerve lesion [10], supported by evidence on electromyography, muscle histology, and electron microscopy [11]. Third, an aberrant regeneration of the oculomotor nerve is associated with intermittent conduction block in the regenerated fibres [12]. However, we found no clinical features of aberrant regeneration. Fourth, the cyclic spasms might be related to the pathophysiology of ocular neuromyotonia [13]. However, the contractions in neuromyotonia are induced by sustained eccentric eye movement and are non-rhythmic. A nuclear site for the lesion is not plausible [5,10,11].

We favor and propose that the lesion(s) in our patient are peripheral, since a nuclear oculomotor lesion causes bilateral superior rectus and levator palpebrae superioris weakness, and this was not seen in our case. The first mechanism proposed above could also be peripheral if the lesion occurs after the transition between central and peripheral myelin or even within the brainstem. However, a central causation in that region would presumably involve the fascicular course of the third nerve before the transitional zone and would likely be associated with other clinical findings.

We cannot exclude additional central mechanisms that could be involved in the pathogenesis, which remains unknown currently. In addition, there are some limitations on anatomical localization based solely on the clinical phenotype.

Management

Treatment for this condition is limited. Surgery to correct ptosis may lead to exposure keratopathy, but strabismus surgery should be considered if desired by the patient or family. Currently, there is no proven treatment for the cyclic pupil spasms [6], although cyclic esotropia, which our patient did not have, can be successfully treated with surgery or botulinum toxin [14,15]. In addition, a case report involving a young adult with long-standing cyclic ptosis stated that a tarsofrontal silicone sling surgery diminished his eyelid cycling, likely through bypassing levator muscle action [9], i.e., the frontalis sling masked the cyclic spasms of the weak levator muscle.

Limitations

Our longitudinal long-term data pertains to a single patient; therefore, our results may not be generalizable. The use of Cardiff cards, LEA symbols, and Snellen VA may make longitudinal comparison of VA inaccurate. Measurements during the autorefraction every 30 seconds may miss short-duration transitions between the two phases. In addition, the observation periods were relatively short, and the number of cycles analyzed in detail is small, which may make the data less precise. As such, the measurements represent snapshots of cycle behavior and not a comprehensive characterization.

## Conclusions

Based on this single case report, we conclude that congenital cyclic oculomotor nerve palsy causes weakness in one or more muscles innervated by the oculomotor nerve, which may unfold sequentially throughout infancy and childhood. The cyclic spasms have a variable age of onset, but they usually start during infancy. The spasms may affect one muscle at onset and progress to involve other muscles over a period of several years. The duration of the full cycle and its two phases is variable across different muscles during any one cycle. In addition, the duration of the full cycle and its two phases is variable during sequential cycles in any one muscle. Furthermore, the duration of the full cycle and its two phases is variable across different muscles at different ages.

## References

[1] McGregor IS (1945). Cyclic oculomotor palsy. J Neurol Neurosurg Psychiatry.

[2] Price DM, Trounce DQ (1973). Cyclic oculomotor paralysis. Arch Dis Child.

[3] Susac JO, Smith JL (1974). Cyclic oculomotor paralysis. Neurology.

[4] Yazici B, Unal M, Köksal M, Ozdek SC (2000). Oculomotor palsy with cyclic spasms: a case report. Orbit.

[5] Loewenfeld IE, Thompson HS (1975). Oculomotor paresis with cyclic spasms. A critical review of the literature and a new case. Surv Ophthalmol.

[6] Chan SC, Li F, Watts P (2026). Congenital cyclic oculomotor palsy and spasms: a review of the literature and presentation of two new cases. J AAPOS.

[7] Nan H, Li R, Zhu L, Wang Q, Wang J, Jing Y (2021). Oculomotor paresis with cyclic spasms in Chinese populations: A review of the Chinese literature and a case report. Pediatr Neurosurg.

[8] Salman MS, Klassen SF, Clark IH (2015). Congenital oculomotor nerve paresis with isolated cyclic pupillary spasms. J Neuroophthalmol.

[9] Bansal S, Das S (2017). Frontalis sling surgery: a treatment modality for cyclic oculomotor nerve palsy. Saudi J Ophthalmol.

[10] Friedman DI, Wright KW, Sadun AA (1989). Oculomotor palsy with cyclic spasms. Neurology.

[11] Kommerell G, Mehdorn E, Ketelsen UP (1985). Oculomotor paralysis with cyclic spasms; electromyographic and electron microscopic indications of chronic peripheral nerve irritation (Article in German). Fortschr Ophthalmol.

[12] Bateman DE, Saunders M (1983). Cyclic oculomotor palsy: description of a case and hypothesis of the mechanism. J Neurol Neurosurg Psychiatry.

[13] Abdulla N, Eustace P (1999). A case of ocular neuromyotonia with tonic pupil. J Neuroophthalmol.

[14] Akyuz Unsal AI, Özkan SB, Ziylan S (2019). Role of botulinum toxin type A in cyclic esotropia: a long-term follow-up. J Pediatr Ophthalmol Strabismus.

[15] Çelik S, Inal A, Ocak OB, Aygıt ED, Akar S, Gökyiğit B (2019). Cyclic strabismus: what measured angle of strabismus should guide surgery?. Strabismus.

